# Okra ameliorates hyperglycaemia in pre-diabetic and type 2 diabetic patients: A systematic review and meta-analysis of the clinical evidence

**DOI:** 10.3389/fphar.2023.1132650

**Published:** 2023-04-03

**Authors:** Kabelo Mokgalaboni, Sogolo Lucky Lebelo, Perpetua Modjadji, Saba Ghaffary

**Affiliations:** ^1^ Department of Life and Consumer Sciences, College of Agriculture and Environmental Sciences, Florida Campus, University of South Africa, Roodepoort, South Africa; ^2^ Non-Communicable Disease Research Unit, South African Medical Research Council, Cape Town, South Africa; ^3^ Department of Public Health, School of Healthcare Sciences, Sefako Makgatho Health Sciences University, Ga-Rankuwa, South Africa; ^4^ Hematology and Oncology Research Center, Tabriz University of Medical Sciences, Tabriz, Iran

**Keywords:** okra, antioxidant, type 2 diabetes, inflammation, hyperglycaemia, pre-diabetes

## Abstract

**Background:** Despite the use of available pharmaceutical drugs, high rates of metabolic diseases and cardiovascular disorders are alarming. This calls for alternative therapies that can attenuate these complications. Therefore, we investigated the beneficial effects of okra on glycaemic control in pre-diabetes and type 2 diabetes mellitus (T2D).

**Methods:** MEDLINE and Scopus were searched for relevant studies. Collected data were analysed using RevMan and reported as mean difference and 95% confidence intervals (CI). Eight studies, including 331 patients with pre-diabetes or T2D, were eligible.

**Results:** Our findings showed that okra treatment reduced the levels of fasting blood glucose: mean difference (MD) = −14.63 mg/dL; 95% CI (-25.25, −4.00, *p* = 0.007); *I* ^*2*^ = 33%, *p* = 0.17 compared to placebo. Glycated haemoglobin, however, did not differ significantly between the groups: MD = 0.01%; 95%CI (-0.51, 0.54, *p* = 0.96); *I* ^*2*^ = 23%, *p* = 0.28.

**Conclusion:** this systematic review and meta-analysis found that okra treatment improves glycaemic control in patients with pre-diabetes or T2D. The findings suggest that okra may be used as a supplemental dietary nutrient, especially in pre-diabetic and T2D patients due to its potential to regulate hyperglycaemia.

## 1 Introduction

Type 2 diabetes mellitus (T2D) remains a public concern in modern life (Zheng et al., 2018), responsible for most cardiovascular diseases (CVD) and metabolic complications (Ma et al., 2022). In particular, an impaired lipid profile, or dyslipidaemia, in T2D is associated primarily with the development of atherosclerosis and a high mortality rate, as seen in T2D patients (Garg et al., 2015; Wengrofsky et al., 2019). Therefore, controlling blood glucose in T2D is important in the prevention of secondary complications associated with T2D, such as atherosclerosis (Hasheminasabgorji and Jha, 2021). Although readily available medicines such as metformin have proven to be beneficial in treating T2D and metabolic conditions by controlling glucose levels (Aroda and Ratner, 2018; Dludla et al., 2021), there is still a continuous rise in the death rate associated with T2D complications.

Similarly, the use of statins to control dyslipidaemia in T2D is well documented (Pinal-Fernandez et al., 2018), and evidence from pre-clinical and clinical studies has shown that statins can result in increased insulin resistance and hyperglycaemia (Biteker et al., 2011; Anyanwagu et al., 2016). Persistent hyperglycaemia in T2D predisposes the patients to the development of other secondary complications. This calls for a natural, effective treatment with fewer side effects targeted towards patients at high risk of developing diabetes or diabetic patients with a high risk of secondary complications.

Therefore, the use of alternative treatments that are easily accessible can be a breakthrough in regulating complications that arise in T2D patients. Thus, other herbal plants with antioxidant activities have gained much attention in scientific research as these have beneficial impacts on oxidative stress and glycaemic control (Adelakun and Oyelade, 2011; Ayodeji et al., 2014; Nasri et al., 2015; Rajendiran et al., 2018). One example has been the use of traditional plants such as okra (*Abelmoschus esculentus L*) due to their antioxidant properties (Adelakun and Oyelade, 2011). The okra plant belongs to the family of Malvaceae, and it is rich in flavonoids, which contribute to its anti-oxidative properties (Liao et al., 2019a). A recent meta-analysis of pre-clinical evidence demonstrated improved insulin sensitivity, lipid profile, and body weight without direct analysis of fasting blood glucose (FBG) and glycated haemoglobin (HbA1c) in the animal model of diabetes (Sereno et al., 2022). Interestingly, a comprehensive review by (Nikpayam et al., 2021) has also demonstrated the beneficial effect of okra on lipid profiles and glycemic parameters; however, this study also analysed evidence from animal models of obesity and T2D in addition to T2D patients.

In contrast to evidence from previous studies, our current meta-analysis has evaluated glycemic control in pre-diabetes and T2D with a major focus on FBG and HbA1c. According to our knowledge, this is the first meta-analysis to critically evaluate evidence from clinical studies exploring the beneficial effects of okra in pre-diabetes and T2D, focusing on direct markers of glycaemic control.

## 2 Methods

The current meta-analysis was conducted and reported according to the PICO (Frandsen et al., 2020) and Preferred Reporting Items for Systematic Reviews and Meta-analyses (PRISMA) guidelines (Page et al., 2021) (Appendix 1), respectively. According to our knowledge, this is the first meta-analysis to explore the beneficial effects of okra in clinical studies.

### 2.1 Question and PICO guideline

This meta-analysis was conducted to address the following question.

1. Do plant-based remedies such as okra improve hyperglycaemia in pre-diabetes and T2D?

Our target population included adult patients with pre-diabetes and T2D, the intervention was any form of okra treatments, and the comparator was pre-diabetic and T2D patients on a placebo, considered a control. The outcomes included hyperglycaemic control, focusing on FBG and HbA1c.

### 2.2 Information sources and literature search

The MEDLINE, through the PubMed search engine and Scopus databases, was systematically searched for relevant studies using the following MesH terms: “Okra”; and its species names; “*Hibiscus Esculentus*”; “*H. Esculentus*”; “*Abelmoschus Esculentus*”; “*Hibiscus sabdariffa Linn,*”; and “type 2 diabetes mellitus”. Additionally, studies evaluating the effect of okra on patients with pre-diabetes, also known as impaired glucose tolerance, were included. The literature search was performed without language restrictions; hence, all languages were considered. The initial search was made on 17 July 2022 and updated on 11 November 2022. All identified studies were evaluated independently by reading the title and abstract to exclude irrelevant studies. This was followed by retrieving the full texts of eligible studies to assess their relevance to the aim of the study.

### 2.3 Study selection

All studies identified through the online databases were saved on the Mendeley desktop and Microsoft Team for all investigators to access after each phase. First, the titles, keywords, and abstracts of all articles obtained from the primary search were screened by two independent investigators (KM and SG). In brief, these two investigators also screened and extracted data from all relevant full texts studies that were retrieved according to eligibility criteria. Disagreement between KM and SG about any study was resolved through discussion and re-evaluation of the study in question, in addition to the independent opinion from the third investigator (PM).

### 2.4 Inclusion and exclusion criteria

All clinical studies conducted on pre-diabetes/T2D patients receiving okra treatment were included. Only studies published in peer-reviewed journals from inception until 11 November 2022 were included. Studies using an animal model of diabetes, those without a control group, and review articles were excluded.

### 2.5 Data extraction risk of bias and quality assessment

Two independent investigators (KM and SG) extracted the following data from all relevant studies: Surname of the first author; year of publication; country, condition, and population; age; gender; the form of okra and dose; duration of intervention; and general findings. The quality and risk of bias were independently assessed using the Cochrane risk-of-bias tool (Julian Higgins et al., 2016). The following domains were evaluated and judged as either low, high, or unclear risk of bias: random sequence generation, allocation concealment, blinding of participants and personnel, blinding of outcome assessment, incomplete outcome data, and selective reporting. Disagreements between investigators (KM and SG) were resolved by discussing and re-evaluating the study or items in dispute.

### 2.6 Statistical analysis

Meta-analysis was considered when two or more studies reported the same outcome; otherwise, a qualitative approach was taken. All quantitative data were analysed using RevMan software (version 5.4; Cochrane Collaboration, Oxford, United Kingdom). Mean difference (MD) and 95% CI were used as all outcomes across the groups were reported using the same unit of measurement. An *I* ^*2*^ (Higgins and Thompson, 2002) and Chi-squared tests were used to assess heterogeneity. Briefly, heterogeneity was interpreted as moderate, low, or no evidence of heterogeneity if *I* ^*2*^ was 70%, 40%, or 0%, respectively. The source of heterogeneity was investigated through subgroup analysis, while a sensitivity test was used to evaluate the stability of our effect size (Mathur and Vander Weele, 2020). On the other hand, a Grading of Recommendations, Assessment, Development, and Evaluations (GRADE) technique was followed to evaluate the certainty of our gathered evidence. A probability value of less than 5% was considered statistically significant.

## 3 Results

**FIGURE 1:**
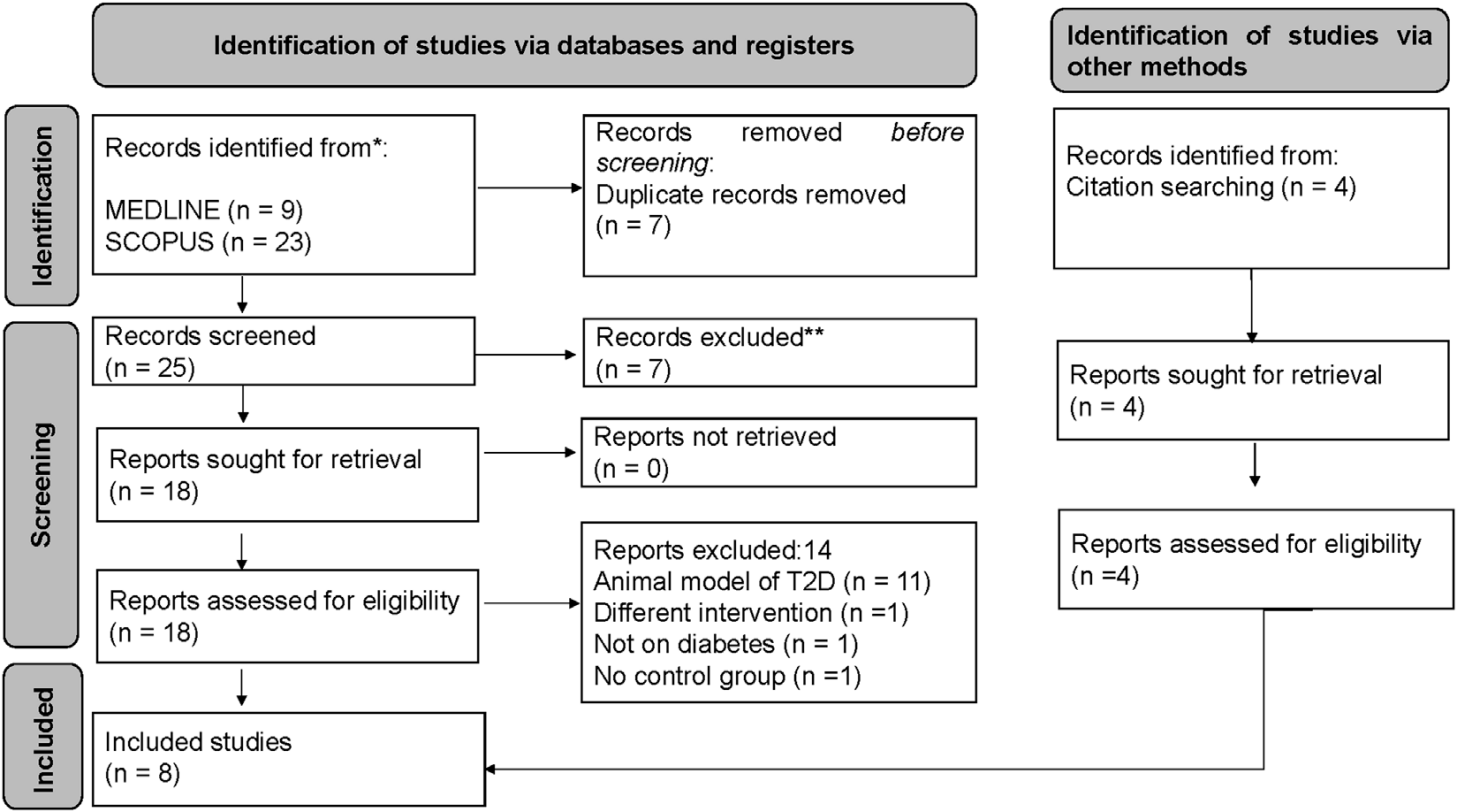
PRISMA flow diagram showing the study screening process.

We identified about nine records from MEDLINE and about 23 records from SCOPUS. About seven records were duplicates from both databases and were excluded from the study. Following an initial screening of the title, abstract, and keywords, seven studies were excluded, three of which did not evaluate okra in pre-diabetes or T2D, and four were reviews. A total of 18 studies were retrieved for eligibility; 11 were pre-clinical studies using an animal model of T2D, one used a different intervention, one was not on pre-diabetes or T2D, and another had no control group. We further searched for relevant studies from the bibliography of eligible studies; interestingly, four additional studies were found to be relevant. Therefore, only eight studies were found relevant and included in the study; however, only seven were included in the meta-analysis due to the data presented (Figure 1). The PRISMA flow diagram was created using the R package and Shiny App (Haddaway et al., 2022). The exact search strategy applied in both databases is presented in Supplementary Table S1 and Supplementary Table S2.

### 3.1 General overview of the included studies

Table 1 shows the general overview of these studies; about eight (Labadnoy et al., 2017; ADAP et al., 2019; Haryati and Rahmawati, 2019; Sarbini et al., 2019; Ziaee et al., 2019; Khodija et al., 2020; Moradi et al., 2020; Saatchi et al., 2022) studies were included, with about 331 patients with pre-diabetes or T2D. One study (Khodija et al., 2020) had participants with T2D and hypercholesterolemia, two had impaired fasting glucose (Labadnoy et al., 2017; ADAP et al., 2019), while others were strictly on T2D (Haryati and Rahmawati, 2019; Sarbini et al., 2019; Ziaee et al., 2019; Moradi et al., 2020; Saatchi et al., 2022). The studies were published between 2017 and 2022, with three studies in Indonesia (Haryati and Rahmawati, 2019; Sarbini et al., 2019; Khodija et al., 2020), three in Iran (Ziaee et al., 2019; Moradi et al., 2020; Saatchi et al., 2022), and two in the Philippines (Labadnoy et al., 2017; ADAP et al., 2019). Different study designs were considered; five were randomised controlled trials (RCTs) (Labadnoy et al., 2017; Sarbini et al., 2019; Ziaee et al., 2019; Moradi et al., 2020; Saatchi et al., 2022); there was one clinical trial (Khodija et al., 2020), and two were quasi-experimental (ADAP et al., 2019; Haryati and Rahmawati, 2019). In all studies, more participants receiving okra treatments were females compared to males, with a male-to-female ratio of 1:2. The participants were aged between 35 and 60. One study was considered to have a short intervention period as it was conducted at intervals of an hour for 3 hours (ADAP et al., 2019), two studies were conducted over 2 weeks (Haryati and Rahmawati, 2019; Khodija et al., 2020), one for 4 weeks (Labadnoy et al., 2017), three for 8 weeks (Sarbini et al., 2019; Moradi et al., 2020; Saatchi et al., 2022), and one for over 12 weeks (Ziaee et al., 2019). Most of the okra was administered in the form of powder or as an immersion.

**TABLE 1:** Overview characteristics of included clinical studies exploring the effects of okra in pre-diabetes and type 2 diabetes.

Authors, year	Study design, country	Population	M: F	Age (Years)	Dose of okra and duration of intervention	Effects on glycaemic control
Labadnoy et al. (2017)	Randomised controlled trials (RCT)	Twenty-four type 2 diabetes (T2D) patients	5:7	54 ± 9.9	A 0.6 g of okra pod powder was used for 4 weeks	No significant changes were observed in fasting blood glucose (FBG) between the okra and placebo groups
	Philippines					
Sarbini et al. (2019)	RCT	Fifty-two T2D patients	7:17	54.08 ± 5.89	A 50 mg of okra extract 2 times a day for 8 weeks	Okra resulted in a significant decrease in FBG levels in T2D
	Indonesia					
ADAP et al. (2019)	QE	Seventeen patients with impaired fasting glucose (IFG)	NR	35	A 200 mg/kg powdered okra seed-peel mixed with the glucose load was consumed for 3 h	Powdered peel of okra decreased FBG levels of IFG patients
	Philippines					
Haryati and Rahmawati (2019)	QE	Thirty T2D patients	4:11	40 to > 60	About 250 mL of okra fruit immersion was given for 2 weeks	Okra significantly decreased FBG.
	Indonesia					
Ziaee et al. (2019)	RCT	Sixty-six T2D patients	12:21	59.2 ± 4.1	A 200 mg hydroalcoholic extract of okra was given 2 times a day for 12 weeks	Okra significantly decreased FBG and glycated haemoglobin (HbA1c)
	Iran					
Khodija et al. (2020)	Clinical trial	Forty T2D patients with hypercholesterolemia	2:22	45–65	About 40 g of okra was given for 2 weeks	Okra significantly decreased FBG in T2D patients
	Indonesia					
Moradi et al. (2020)	RCT	Sixty T2D patients	9:21	54.26 ± 7.62	About 10 g of okra powder blended with 150 g of conventional yogurt was given twice for 8 weeks	Okra significantly decreased FBG without a significant effect on HbA1c in T2D
	Iran					
Saatchi et al. (2022)	RCT	Hundred and twenty T2D patients	23:26	57.7 ± 9.7	About 1,000 mg of okra whole fruit powder was given orally as a capsule every 6 h for 8 weeks	Okra significantly decreased FBG and HbA1c in T2D
	Iran					

*T2D, type diabetes; FBG, fasting blood glucose; QE, quasi-experimental; RCT, randomised controlled trials; HbA1c, glycated haemoglobin; M, male; F, female.

### 3.2 The risk of bias and quality assessment

Figure 1 in the Supplementary File S1 presents the risk of bias across the included studies. Four (Sarbini et al., 2019; Ziaee et al., 2019; Moradi et al., 2020; Saatchi et al., 2022) studies specified randomisation methods used in their respective studies. Briefly, they were judged as low risk except for three studies (Labadnoy et al., 2017; ADAP et al., 2019; Haryati and Rahmawati, 2019; Khodija et al., 2020) which were classified as high risk as methods were not specified. One study indicated no randomisation method was performed (Labadnoy et al., 2017). For allocation concealment, four studies (Sarbini et al., 2019; Ziaee et al., 2019; Moradi et al., 2020; Saatchi et al., 2022) reported that the allocations of intervention were concealed from the researcher and participants until data analyses were completed and thus were judged as having a low risk of bias. Two other quasi-experimental studies (Haryati and Rahmawati, 2019; Khodija et al., 2020) did not provide any information regarding the allocation method and were judged as having a high risk of bias. Two other studies (Labadnoy et al., 2017; ADAP et al., 2019) were judged as unclear risks as the authors did not indicate if there was allocation concealment. In five (Labadnoy et al., 2017; Sarbini et al., 2019; Ziaee et al., 2019; Moradi et al., 2020; Saatchi et al., 2022) studies, participants and personnel were double-blinded. Three studies (ADAP et al., 2019; Haryati and Rahmawati, 2019; Khodija et al., 2020) did not provide any information regarding this domain and thus were considered an unclear risk of bias. For blinding of the outcome, four studies (Sarbini et al., 2019; Ziaee et al., 2019; Moradi et al., 2020; Saatchi et al., 2022) were judged as low-risk, and another four studies (Labadnoy et al., 2017; ADAP et al., 2019; Haryati and Rahmawati, 2019; Khodija et al., 2020), were judged as unclear as no information was provided on this domain. For the incomplete outcome data domain, all studies were considered a low-risk bias; for example, five (Labadnoy et al., 2017; Sarbini et al., 2019; Ziaee et al., 2019; Moradi et al., 2020; Saatchi et al., 2022) reported the number of patients who did not complete the treatment regimen due to unwillingness to continue with the study or loss of follow-up. In contrast, the other three studies (ADAP et al., 2019; Haryati and Rahmawati, 2019; Khodija et al., 2020) did not have any loss of patients throughout the study. In terms of the selective reporting domain, all studies (Labadnoy et al., 2017; ADAP et al., 2019; Haryati and Rahmawati, 2019; Sarbini et al., 2019; Ziaee et al., 2019; Khodija et al., 2020; Moradi et al., 2020; Saatchi et al., 2022) did not have a protocol published. Therefore, it was difficult to judge whether there was a diversion from originally planned outcome measures and analysis; thus, they were judged as having an unclear risk of bias for this domain.

### 3.3 Results of our quantitative (meta-analysis) analysis of included studies

#### 3.3.1 Effects of okra treatment on glycaemic control in type 2 diabetes patients

**FIGURE 2:**
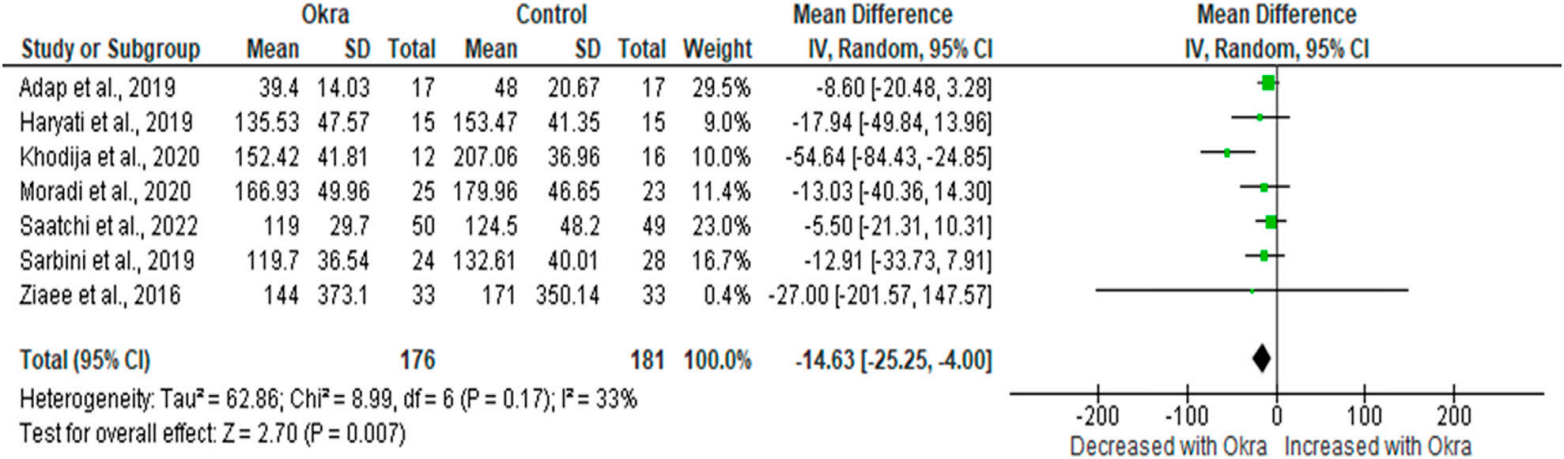
The effect of okra treatment on fasting blood glucose in type 2 diabetic patients. Results presented as mean difference, 95% confidence intervals. A *p*-value of 5% or less is considered statistically significant.

In this study, FBG and HbA1c were considered the main glycaemic parameters. Therefore, about eight clinical studies reported the effects of okra on the level of FBG. However, due to a lack of data from one study (Labadnoy et al., 2017), a meta-analysis was conducted on only seven studies (ADAP et al., 2019; Haryati and Rahmawati, 2019; Sarbini et al., 2019; Ziaee et al., 2019; Khodija et al., 2020; Moradi et al., 2020; Saatchi et al., 2022). Pre-diabetes and T2D patients receiving okra treatment had decreased FBG levels compared to the group on the placebo [MD = −14.63 mg/dL 95%CI (−25.25, −4.00); *p* = 0.007]. However, these studies had minimal heterogeneity, as demonstrated by (*I* ^*2*^ = 33%, *p* = 0.17) (Figure 2).

#### 3.3.2 Effect of okra on glycated haemoglobin (HbA1c)

**FIGURE 3:**
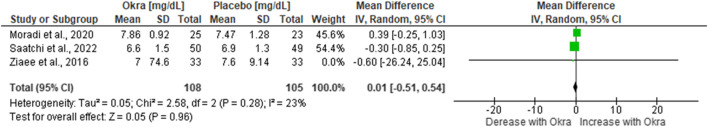
Effects of okra on glycated haemoglobin (HbA1c) in type 2 diabetes patients.

Glycated haemoglobin is a form of haemoglobin that is chemically linked to blood and thus used to measure the level of blood glucose. Three studies (Ziaee et al., 2019; Moradi et al., 2020; Saatchi et al., 2022) explored the benefits of okra on HbA1c in pre-diabetes and T2D patients. The analysed data from these three studies showed no statistically significant difference in HbA1c among pre-diabetes or T2D patients on okra treatment compared to the group on the placebo [MD = 0.01%; 95%CI (−0.51, 0.54, *p* = 0.96)]. Interestingly, these studies revealed a low level of heterogeneity (*I* ^*2*^ = 23%, *p* = 0.28) (Figure 3).

### 3.4 Subgroup analysis of okra treatment on FBG

**FIGURE 4:**
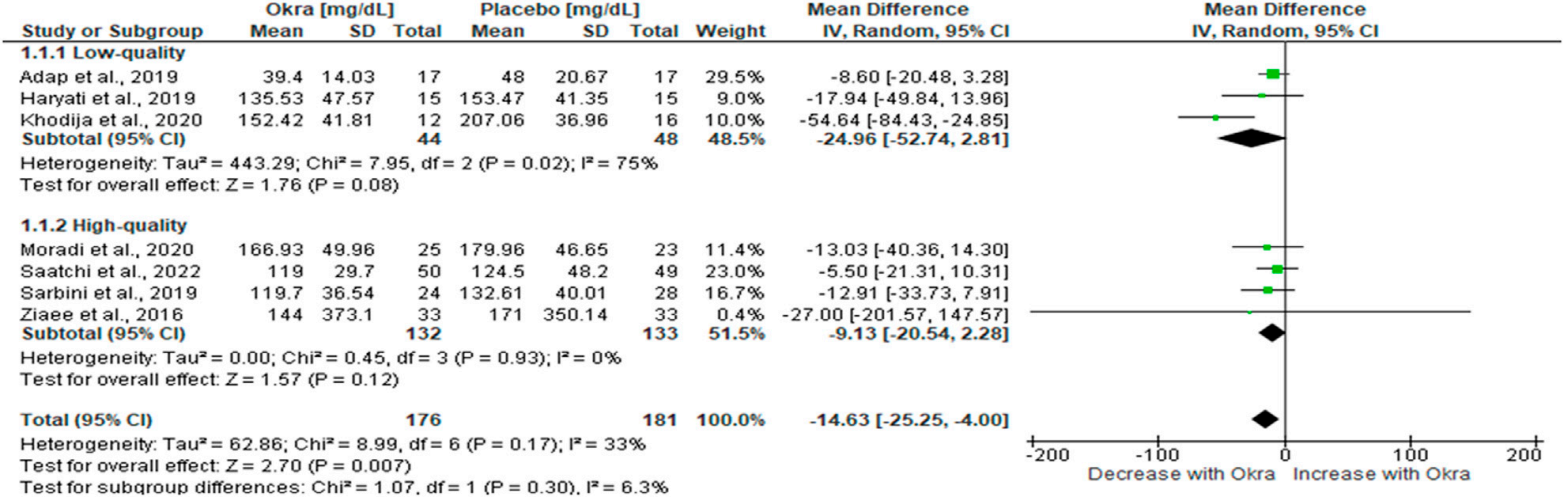
Subgroup analysis of FBG according to the quality of the included studies.

Heterogeneity remains a challenge in meta-analysis; this can arise due to methodological differences, quality of studies, risk of bias, and other confounding factors. Therefore, we performed subgroup analysis in this study due to the heterogeneity observed among studies evaluating okra’s effects on FBG. This was performed according to the study designs and the quality of the study. It was evident that heterogeneity might not be due to the study design as the same results were observed [MD = −14.63%, 95%CI (−25.25, −4.00); *p* = 0.007] with *I* ^*2*^ = 75.8% (Supplementary Figure S2). Similarly, when subgroup analysis was performed based on the quality of studies, we observed the same results; however, this time with a very minimal level of heterogeneity (*I* ^*2*^ = 6.3%) (Figure 4). Furthermore, our analysis revealed that studies with high quality, as per Cochrane guidelines, had a decreased FBG [MD = −9.13 mg/dL, 95%CI (−20.54, 2.28) (Figure 4]. Interestingly, this was accompanied by evidence of no heterogeneity (*I* ^*2*^ = 0%) (Figure 4). Thus, this leads us to conclude that heterogeneity in this meta-analysis was due to studies with low quality (ADAP et al., 2019; Haryati and Rahmawati, 2019; Khodija et al., 2020).

### 3.5 Sensitivity analysis

To explore the possible effect of a single study on the overall effect estimate and how robust our effect size is, we conducted a sensitivity analysis through one study exclusion approach. When a study with a smaller sample size was excluded, the effect estimates changed significantly. Following the exclusion of a study by Khodija et al. (2020) due to the small sample size, the FBG overall effect size changed significantly from the original effect to [MD = −9.44%, 95%CI (−17.41,−1.47), *p* = 0.02], and this was coupled with evidence of no heterogeneity (*I* ^*2*^ = 0%, *p* = 0.98) (Supplementary Figure S3). Similarly, a sensitivity analysis was performed for HbA1c by excluding the same study due to a smaller sample size, and we observed a significant change in effect size compared to the original one [MD = −0.30, 95%CI (−0.85,0.25), *p* = 0.29]; however, this was statistically not significant. Of interest among these studies was no evidence of heterogeneity (*I* ^*2*^ = 0%, *p* = 0.98) (Supplementary Figure S4).

### 3.6 Publication bias and GRADING of evidence

Due to a limited number of studies, publication bias was not visualised graphically in the current meta-analysis. However, it is important to note that, even though publication bias was not conducted, the evidence gathered was published from various countries and showed consistent findings; this may rule out suspicions of publication bias based on the origin of the studies. Moreover, we used the Grading of Recommendations, Assessment, Development, and Evaluations (GRADE) technique to evaluate the certainty of our evidence, and the results are presented as a summary of findings (SoF) table in Supplementary Table S2. Our findings demonstrated a relatively moderate level of certainty in FBG, while there was a high level of certainty in HbA1c.

## 4 Discussion

Diabetes mellitus (DM) is an increasing public health burden, with an estimated prevalence of 25% in the global diabetic population (Zheng et al., 2018). Despite the availability of pharmaceutical drugs against T2D, there is still an increasing number of secondary complications associated with T2D and a concerning mortality rate among pre-diabetic and T2D patients. This calls for more research into new and effective therapies with fewer side effects. Natural plants and dietary compounds are increasingly being explored for their unique antioxidant and hyperglycemic properties in diabetic patients (Graf et al., 2010; Nasri et al., 2015; Unuofin and Lebelo, 2020). As a result, several dietary compounds have gained special research interest owing to their ameliorative and antioxidant effects on metabolic and cardiovascular complications associated with diabetes (Nasri et al., 2015; Unuofin and Lebelo, 2020). This is partially due to the rate of affordability, few to no side effects, and better compliance of patients on herbal remedies. Therefore, it remains crucial to understand the beneficial effects and mode of action of these dietary compounds with antioxidant properties, such as okra, amongst pre-diabetes and T2D patients. Pre-diabetic patients are generally at high risk of developing T2D, especially if they are not treated; therefore, they need regular blood glucose monitoring to ensure their blood glucose is fully controlled. Several pre-clinical studies have explored the impact of this plant in animal models of obesity (Fan et al., 2014) and T2D (Huang et al., 2018; Yaradua et al., 2018; Husen et al., 2019; Unuofin and Lebelo, 2020). However, the results are still inconsistent in pre-clinical studies (Prom-In et al., 2020).

To the best of our knowledge, this is the first meta-analysis to explore and critically discuss the evidence from clinical studies on the impact of okra treatment on glycaemic control in pre-diabetic and T2D patients. A detailed literature search was performed, and about 8 clinical studies exploring the effect of okra in pre-diabetic and T2D patients were identified. We found that okra treatment in pre-diabetic and T2D patients has beneficial effects on glycaemic control, as demonstrated by a significant reduction in the level of FBG. These findings are consistent with the results reported by previous researchers (Khatun et al., 2011; Majd et al., 2018; Nguekouo et al., 2018; Husen et al., 2020; Uadia et al., 2020; Wu et al., 2020; Yan et al., 2020; Tyagita et al., 2021; Haque et al., 2022). One of the proposed mechanisms that okra targets in reducing FBG is the stimulation of hepatic glycogen synthesis and pancreatic islet regeneration, thus leading to increased insulin secretion and subsequent delay in the intestinal absorption of glucose. This is accompanied by a significant glucose dialysis retardation index and increased glucose adsorption capacity (Abbas et al., 2018). Other implicated mechanisms in the anti-hyperglycemic effect of okra include improvement of glucose homeostasis and β-cells impairment through a peroxisome proliferator-activated receptors (PPAR)-dependent pathway (Majd et al., 2018). PPAR is a group of ligand-activated transcription factors that are involved in the regulation of blood glucose levels. It is common knowledge that oxidative stress, characterised by increased reactive oxygen species (ROS), is implicated in the development of T2D by inducing pancreatic β-cells dysfunction, insulin resistance, and impaired glucose tolerance (Bhatti et al., 2022). Interestingly, the high content of phenols in okra improves insulin resistance and the function of pancreatic β-cells by reducing ROS (Patel and Barnes, 2010). It is important to note that okra is an antioxidant-rich in flavonoids, quercetin, polyphenols, and vitamins A and C (Xia et al., 2015) and has been widely researched in T2D patients and animal models of T2D (Liao et al., 2019b; 2019a; Peng et al., 2019; Moradi et al., 2020; Saatchi et al., 2022).

Moreover, polyphenols act as an agonist for PPAR-γ ligands, thus ameliorating hyperglycaemia (Patel and Barnes, 2010). This explains the growing interest in learning more about the therapeutic benefits of natural antioxidants like okra as an alternative dietary treatment for patients with pre-diabetes and T2D. Previous research indicates that the consumption of a polyphenol-rich diet may lower the risk of CVD by ameliorating oxidative stress (Tressera-Rimbau et al., 2017). Consistent with the findings in this study, a recent systematic review reported the anti-hyperglycaemic effects of okra in animal models of T2D (Saatchi et al., 2022). The evidence currently synthesised in this meta-analysis suggests the potential beneficial effects of okra in improving hyperglycaemia in pre-diabetic and T2D patients.

In contrast to our findings, a study by Labadnoy et al. (2017) showed no significant effect of okra in reducing FBG compared to the group on placebo. Interestingly, the authors showed a significant intergroup decrease in FBG when pre- and post-okra-treatment results were compared. Usually, pre-diabetic patients present with high glucose levels above normal ranges; however, they are not high enough to be classified as T2D. For instance, a study by Akinnuga et al. (2022) has shown that FBG is elevated in pre-diabetes rats compared to non-pre-diabetes rats. However, there was no significant difference in FBG; surprisingly, they also reported a significant increase in HbA1c. Even though okra treatment showed no significant effect on HbA1c, there was a moderate decrease in its levels after we conducted a sensitivity analysis following okra treatment compared to the control group (Supplementary Figure S4). T2D patients are predisposed to various complications, ranging from cardiovascular (Mokgalaboni et al., 2020; Dludla et al., 2022), to immune system (Daryabor et al., 2020), and even reproductive complications (Maresch et al., 2018). Therefore, it is important to manage pre-diabetic and T2D patients’ glucose levels and related parameters to prevent the development of later secondary complications. The anti-hyperglycemic effect of okra has been seen in different animal models of T2D, which corroborates our current findings. For example, previous systematic reviews conducted in an adequate number of pre-clinical studies have shown that the extracts of okra or its various fractions have a significant effect on FBG and HbA1c when given as a short or long treatment in animal models of T2D, as demonstrated by its lowering effects (Nikpayam et al., 2021; Sereno et al., 2022).

Interestingly, the beneficial effects of okra in pre-clinical studies seem to be reproduced in clinical studies, as shown by reduced FBG (ADAP et al., 2019; Haryati and Rahmawati, 2019; Sarbini et al., 2019; Ziaee et al., 2019; Khodija et al., 2020; Moradi et al., 2020; Saatchi et al., 2022), hence suggesting the anti-hyperglycaemic potential of this plant-based remedy in T2D. Although the effects of okra on HbA1c in pre-clinical and clinical studies are contradictory, this might be due to design, administered dosage, or even the form or okra part used. Several pre-clinical studies on the anti-hyperglycaemic properties of okra, focusing on FBG and HbA1c, have yielded promising results.

### 4.1 Strengths and limitations

This is the first meta-analysis to explore the direct effect of okra treatment on glycaemic control in pre-diabetic and T2D patients using clinical evidence. Additionally, the methods used by the independent investigators in the study selection and data extraction processes, the risk of bias, and the quality of evidence assessment were vigorous. Of interest is the certainty of analysed evidence, evaluated using a GRADING technique and was found to be moderate for FBG and high for HbA1c (Supplementary Table S2). Major limitations noted in this study include the different formulations and the dosage of okra, different parts of the okra plant and different extraction methods used, the period of intervention, and study designs. This could explain the notable moderate levels of statistical heterogeneity in this study. However, subgroup and sensitivity analyses were performed to find the source of heterogeneity and evaluate the stability of our effect size. With the limited number of clinical studies (8), the results and conclusions drawn from this study must be interpreted with caution, as only a few clinical studies have been conducted to explore the effects of okra in pre-diabetes and T2D. Lastly, the evidence analysed in this study has proven to be of low quality since there was poor methodological quality in three (38%) studies due to the lack of blinding of participants and personnel, thus subjecting the findings to the risk of bias.

### 4.2 Conclusion

This study is the first meta-analysis to evaluate the effect of okra on glycaemic control in pre-diabetic and T2D patients, and our findings confirm the potential beneficial effects of okra on hyperglycaemia amongst these groups of patients. This is demonstrated by an improved FBG following treatment with okra. Despite the limitation of clinical evidence, the direct beneficial effect of okra as an anti-hyperglycaemic remedy has been demonstrated, and this may protect pre-diabetic and T2D patients against associated secondary complications such as atherosclerosis and related CVDs. Moreover, evidence from pre-clinical studies has also suggested that okra may protect against diabetes-related complications by ameliorating hyperglycaemia and oxidative stress. Therefore, additional long-term clinical trials with adequate sample sizes and improved methodological quality are required to directly examine and validate the effect of okra on the management of T2D by focusing on different glycaemic control parameters, such as FBG, HbAlc, insulin sensitivity, and insulin levels. This can also help to prevent or manage associated CVD complications.

Additionally, the trials should specify the methods followed in the extraction of okra and determine the precise dose and duration of intervention that can provide effective benefits. Most importantly, future clinical trials should be devoid of bias by adhering to the principles of randomisation and blinding and avoiding selection and reporting bias. Since this is the first meta-analysis to explore the antioxidant effects of okra in pre-diabetes and T2D patients, it can also be used as a starting point for future research to highlight and educate the general public about the benefits, adverse effects, and safety measures to be taken when using herbal medicine derived from okra.

## Data Availability

The original contributions presented in the study are included in the article/Supplementary Material, further inquiries can be directed to the corresponding authors.

## Abbreviations

CI, confidence interval; CVD, cardiovascular disease; DM, diabetes mellitus; FBG, fasting blood glucose; GRADE, Grading of Recommendations, Assessment, Development, and Evaluations; HbA1c, glycated haemoglobin; HDL, high-density lipoprotein; LDL, low-density lipoprotein; MD, mean difference; PPAR, peroxisome proliferator-activated receptors; ROS, reactive oxygen species; T2D, type 2 diabetes; TC, total cholesterol.
