# Supplementary material for: Okra ameliorates hyperglycaemia in pre-diabetic and type 2 diabetic patients: A systematic review and meta-analysis of the clinical evidence
Source: Front Pharmacol. 2023 Apr 3;14:1132650. doi: 10.3389/fphar.2023.1132650 (PMC10107009; doi:10.3389/fphar.2023.1132650)
Supplement: Supplementary file 2 [file DataSheet1.docx]

**Supplementary Material**

**Okra ameliorates hyperglycaemia in pre-diabetic and type 2 diabetic patients: A systematic review and meta-analysis of clinical evidence.**

Kabelo Mokgalaboni (MSc)^1*^, Sogolo Lucky Lebelo (PhD)^1^, Perpetua Modjadji (PhD, DrPH)^2,3^, Saba Ghaffary (PhD)^4*^

^1^Department of Life and Consumer Sciences, College of Agriculture and Environmental Sciences, University of South Africa, Florida Campus, South Africa.

^2^Non-Communicable Disease Research Unit, South African Medical Research Council, Cape Town 7505, South Africa

^3^Department of Public Health, School of Health Care Sciences, Sefako Makgatho Health Sciences University, 1 Molotlegi Street, Ga-Rankuwa 0208, South Africa

^4^Hematology and Oncology Research Center, Tabriz University of Medical Sciences, Tabriz, Iran.

Emails: [mokgak@unisa.ac.za](mailto:mokgak@unisa.ac.za); [lebelol@unisa.ac.za](mailto:lebelol@unisa.ac.za) ; [perpetua.modjadji@mrc.ac.za](mailto:perpetua.modjadji@mrc.ac.za) ; [ghaffarys@tbzmed.ac.ir](mailto:ghaffarys@tbzmed.ac.ir) and [saba_gh_64@yahoo.com](mailto:saba_gh_64@yahoo.com)

***Corresponding author:**

Mokgalaboni Kabelo (MSc), Email: [mokgak@unisa.ac.za](mailto:mokgak@unisa.ac.za)

Department of Life and Consumer Sciences, College of Agriculture and Environmental Sciences, University of South Africa, Florida Campus, 1710, South Africa

Calabash Building, Office no 02-047

Tel: +27114713000

**Table 1:** Search strategy adapted on PubMed on 17 July 2022 and updated again on 11 November 2022.

| **MeSH terms** | **Search** | **Records** |
| --- | --- | --- |
| 1. *H. Esculentus* | *H. Esculentus*[MeSH Terms] | 0 |
| 1. *Hibiscus Esculentus* | *Hibiscus Esculentus*[MeSH Terms] | 424 |
| 1. *Okra* | Okra[MeSH Terms] | 424 |
| 1. *Abelmoschus Esculentus* | *Abelmoschus Esculentus*[MeSH Terms] | 424 |
| 1. *Hibiscus sabdariffa Linn* | *Hibiscus sabdariffa Linn*[MeSH Terms] | 0 |
| 1. Type 2 diabetes mellitus | type 2 diabetes mellitus[MeSH Terms] | 163137 |
| 1. 1,2,3,4,5,6 | (((((((*H. Esculentus*[MeSH Terms]) OR (*H. Esculentus*[MeSH Terms])) OR (*Hibiscus Esculentus*[MeSH Terms])) OR (Okra[MeSH Terms])) OR (*Abelmoschus Esculentus*[MeSH Terms])) OR (*Hibiscus sabdariffa Linn*[MeSH Terms])) OR (*Hibiscus sabdariffa Linn*[MeSH Terms])) AND (type 2 diabetes mellitus[MeSH Terms]) | 9 |

**Table 2:** Search strategy applied on Scopus on the 17 July and updated on 11 November 2022.

| **Search terms** | **Records** |
| --- | --- |
| TITLE-ABS-KEY ( "Okra" OR "*H. Esculentus*" OR "*Hibiscus Esculentus*" OR "*Abelmoschus Esculentus*" OR "*Hibiscus sabdariffa Linn*" AND "type 2 diabetes" ) | 23 |


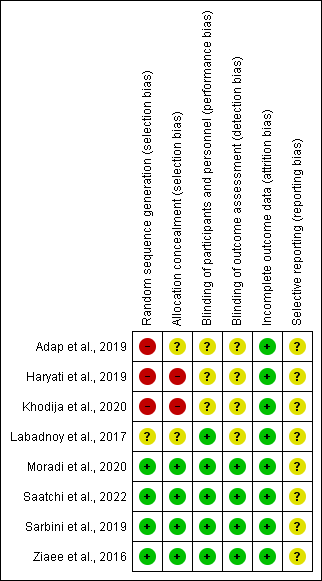


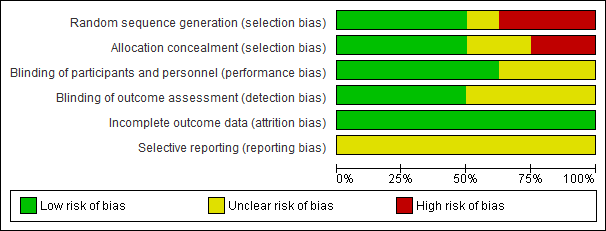


**Figure 1:** Risk of bias summary: review authors' judgments about each risk of bias item for each included study.


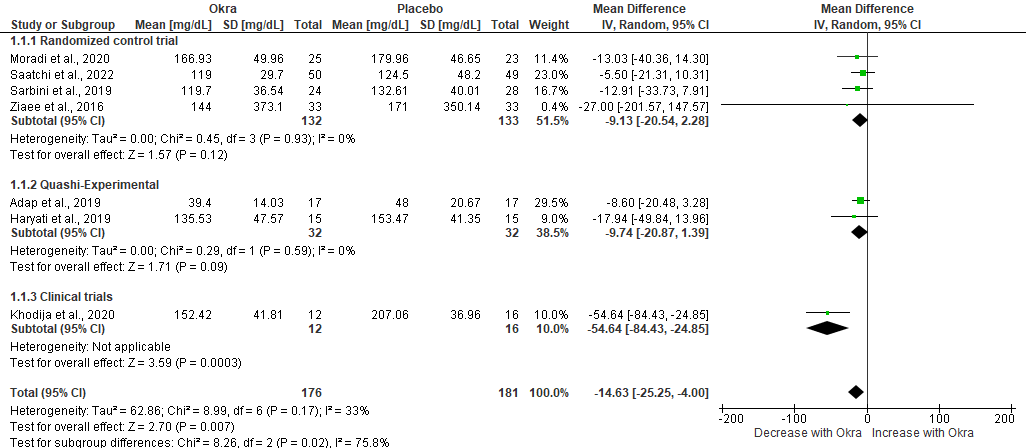


**Figure 2:** Subgroup analysis according to study design, the effect of okra on fasting blood glucose in pre-diabetes and T2D patients.


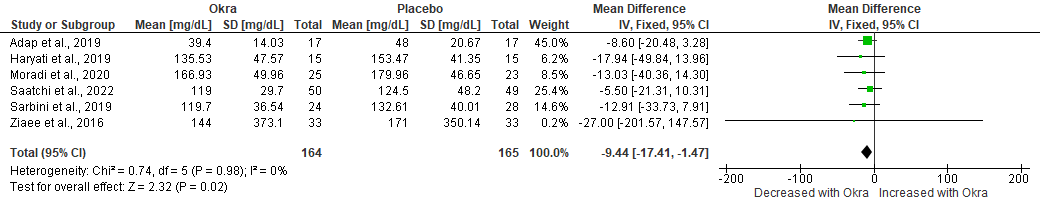


**Figure 3:** Sensitivity analysis, the effect of okra on fasting blood glucose in pre-diabetes and T2D patients.


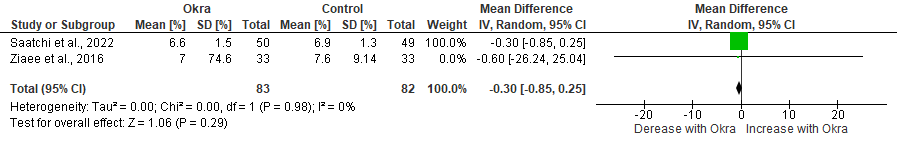


**Figure 4:** Sensitivity analysis, the effect of okra on glycated haemoglobin in type 2 diabetes patients.

**Table 3:** Summary of findings table(SoF) according to GRADING.

|  | | | | | |
| --- | --- | --- | --- | --- | --- |
| **Patient or population:** [Type 2 diabetes & pre-diabetes], Intervention: Okra, Comparison: Placebo | | | | | |
| **Outcomes** | **№ of participants (studies) Follow-up** | **Certainty of the evidence (GRADE)** | **Relative effect (95% CI)** | **Anticipated absolute effects** | |
|  |  |  |  | **Risk with placebo** | **Risk difference with Statins** |
| Fasting blood glucose (FBG) assessed with: mg/dL | 357 (4 RCTs, 2QE, 1C) | ⨁⨁⨁◯ Moderate^a,b,c,d^ | - | The mean FBG ranged from **48-207.06** mg/dL | MD -**14.63 mg/dL**  **lower** (-25.25 lower to -4 lower) |
| Glycated haemoglobin (HbA1c) assessed with: % | 213 (3 RCTs) | ⨁⨁⨁⨁ High^e,f^ | - | The mean HbA1c ranged from **6.9-7.6** % | MD **0.01 % higher** (-0.51 lower to 0.54 higher) |
| ***The risk in the intervention group** (and its 95% confidence interval) is based on the assumed risk in the comparison group and the **relative effect** of the intervention (and its 95% CI).  **CI:** confidence interval; **MD:** mean difference; **QE:** Quasi-Experimental; **C:** Clinical trial | | | | | |
| **GRADE Working Group grades of evidence** **High certainty:** we are very confident that the true effect lies close to that of the estimate of the effect. **Moderate certainty:** we are moderately confident in the effect estimate: the true effect is likely to be close to the estimate of the effect, but there is a possibility that it is substantially different. **Low certainty:** our confidence in the effect estimate is limited: the true effect may be substantially different from the estimate of the effect. **Very low certainty:** we have very little confidence in the effect estimate: the true effect is likely to be substantially different from the estimate of effect. | | | | | |

**Explanations**

a. Two quasi-experimental studies were classified as high risk in two domains according to the Cochrane risk of bias guideline, random sequence generation, and allocation concealment, respectively. In contrast, one clinical study was at risk of bias in terms of random sequence generation.

b. Studies show a minimal level of heterogeneity with *I*^2^ test of 33%.

c. As the number of studies analysed was less than 10, visualising publication bias was not done.

d. Not all studies were RCT, two were Quasi-Experimental, and one was a clinical trial.

e. No risk of bias was noted as per independent investigators through the use of the Cochrane risk of bias tool.

f. The minimal level of heterogeneity was observed (*I*^2^ test of 23%), and this was reduced to zero following sensitivity analysis by excluding a study with a small sample size.
